# LncRNA UCA1 promotes tumor metastasis by inducing miR-203/ZEB2 axis in gastric cancer

**DOI:** 10.1038/s41419-018-1170-0

**Published:** 2018-11-21

**Authors:** Pihai Gong, Fengchang Qiao, Huazhang Wu, He Cui, Yiping Li, Ying Zheng, Menghan Zhou, Hong Fan

**Affiliations:** 1 Department of Medical Genetics and Developmental Biology, Medical School of Southeast University, The Key Laboratory of Developmental Genes and Human Diseases, Ministry of Education, Southeast University, Nanjing, China; 2 School of Life Science, Southeast University, Nanjing, China; 3 Department of Pathophysiology, Medical School of Southeast University, Nanjing, China

## Abstract

Increasing studies showed that long-noncoding RNAs (lncRNAs) play important roles in the biological processes, including cancer initiation and progression. However, little is known about the exact role and regulation mechanism of lncRNA UCA1 during the progression of gastric cancer (GC). In this study, we found that UCA1 was aberrantly elevated in gastric cancer tissues, and was significantly associated with lymph node metastasis and TNM stage. In vivo and in vitro, enforced UCA1 level promoted cell migration and invasion of GC cell. Depleted UCA1 expression level attenuated the ability of cell migration and invasion in GC. And then, we detected that expression level of ZEB2, a transcription factor related to tumor metastasis, was regulated by UCA1 in GC cells. miR-203 targets and suppresses to ZEB2 expression. Furthermore, we found that UCA1 could directly interact with miR-203 and lead to the release of miR-203-targeted transcripts ZEB2. Herein, we revealed the novel mechanism of UCA1 on regulating metastasis-related gene by sponge regulatory axis during GC metastasis. Our findings indicated that UCA1 plays a critical role in metastatic GC by mediating sponge regulatory axis miR-203/ZEB2. To explore function of UCA1-miR-203-ZEB2 axis may provide an informative biomarker of malignancy and a highly selective anti-GC therapeutic target.

## Introduction

Gastric cancer (GC) has the fourth highest diagnosis rate and the third highest mortality among all the cancers throughout the world^1,2^. Cancer metastasis is the leading cause of death in patients with GC, and is mediated by a multistep process referred to as the metastatic cascade, which includes local invasion and migration, angiogenesis, epithelial-mesenchymal transition (EMT), and intravasation^3,4^. Recently, an important class of metastasis-related RNA molecules has attracted more attention, and transcriptomics analysis has underlined the prevalence and functions of long-noncoding RNAs (lncRNAs) in several human cancers^5,6^.

The lncRNA UCA1, located on the human chromosome 19p13.12, was first identified as an oncogene in bladder cancer and showed extensive regulatory functions in cell proliferation^7–10^, invasion^8^, metastasis^11^, apoptosis^12–14^, metabolism^15^, survival^8^, radiosensitivity, and chemoresistance^16,17^. These studies highlight the potential of lncRNA UCA1 as a diagnostic and prognostic biomarker and a therapeutic target in malignant tumors. Some research groups have reported that UCA1 expression was correlated with gastric cancer metastasis when they analyzed clinical information; for example, Zheng et al.^18^ reported that high-UCA1 expression was more frequently detected in advanced TNM stages. Gao et al.^19^ reported that UCA1 expression levels correlated with lymph node metastasis. Zuo et al.^20^ reported that TGF-β1 could induce lncRNA UCA1 upregulation, promoting gastric cancer invasion and migration. These evidences indicate lncRNA UCA1 play an important role during cancer progression. However, the underlying molecular mechanisms in migration of gastric cancer cells are still largely unknown.

Previous studies showed that lncRNAs could cooperate with mRNAs, miRNAs, or proteins to mediate tumorigenesis. lncRNA-ATB increase the stability of IL-11 mRNA and induces autocrine induction of IL-11 to activate IL/STAT3 signaling^21^. Several lncRNAs interact with EZH2, a crucial component of PRC complexes, contributes to guide the complexes to the target loci^22–26^. Notably, along with the development of lncRNA structural analysis, lncRNAs have been found to interact with miRNA, which can play important roles in the diagnosis, treatment, and prognosis for cancer patients, suggesting that lncRNAs may be valuable targets in human cancers.

In this study, we revealed that UCA1 is critical for the progression of GC. The expression level of UCA1 is significantly increased and is closely associated with lymph node metastasis, which is consistent with the TCGA database. Further analyses found that upregulated UCA1 promotes cell migration and invasion by UCA1–miR-203–ZEB2 axis. Together, understanding of the exact functions of UCA1 in the invasion–metastasis cascade could provide new insights into the related clinical difficulties of GC.

## Results

### UCA1 expression level is elevated in tumor tissues of GC patients

**Fig. 1:**
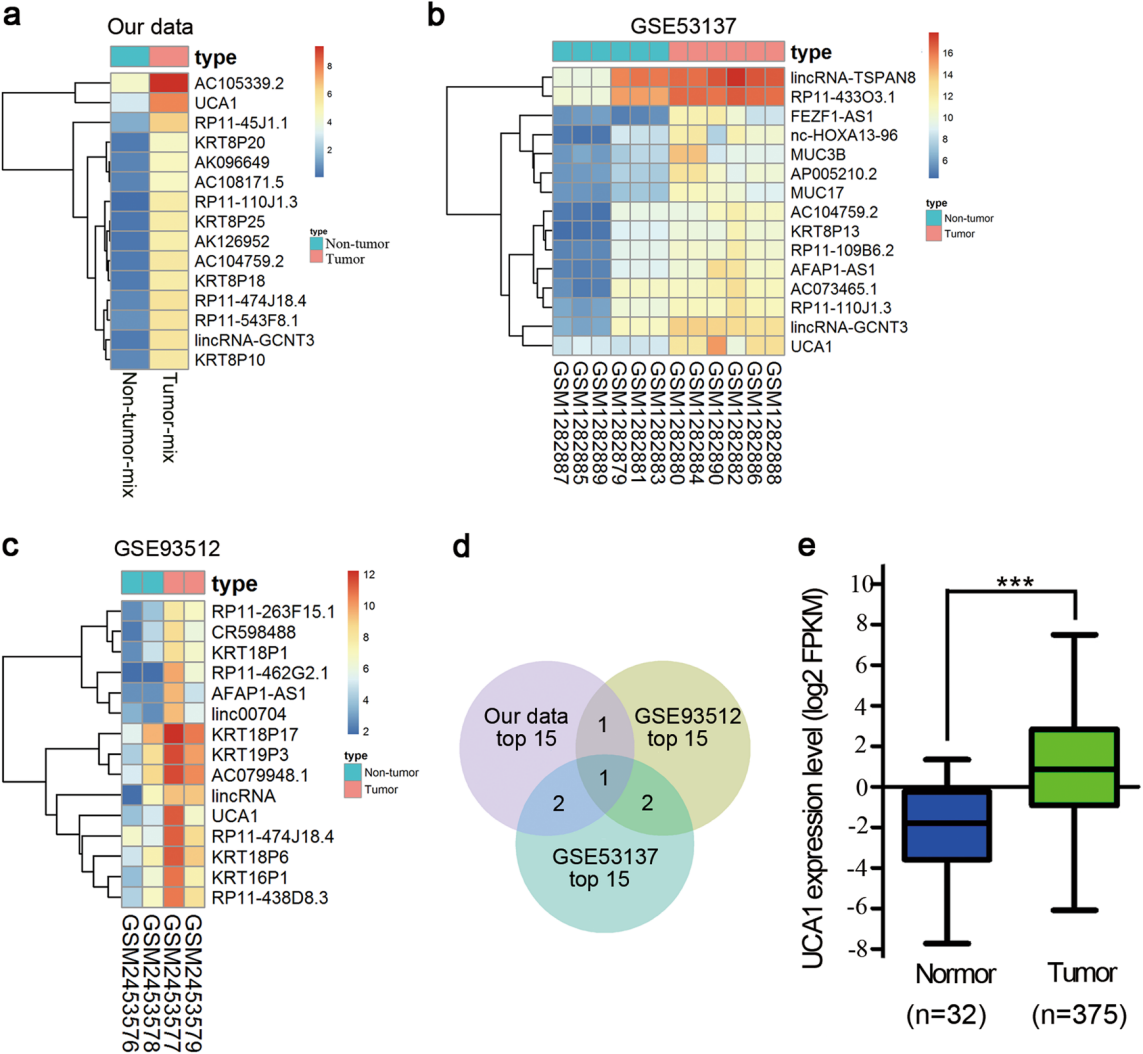
Heat map of lncRNAs whose expression is significantly upregulated. **a** Heat map of the top 15 differentially expressed lncRNAs (fold change > 2; *p* *<* 0.05) in our previous microarray. **b** and **c** Heat map of the top 15 differentially expressed lncRNAs (fold change > 2; *p* *<* 0.05) in GSE53137 and GSE93512. **d** Venn diagram analysis of upregulated lncRNAs shared among our previous microarray data, GSE53137 and GSE93512. **e** UCA1 expression level was elevated in gastric cancer tissues compared with normal tissues, shown from the TCGA data. ***p *<* 0.001 (unpaired Student’s *t*-test)

To identify the lncRNA expression profile in GC, the Arraystar Human lncRNA Microarray was used in five paired GC and their adjacent non-tumor tissues. A total of 3346 downregulated genes and 1853 upregulated genes (fold change ≥ 2) were observed. The top 15 differentially expressed lncRNAs are shown by the heat map (Fig. 1a). Simultaneously, we downloaded two independent datasets, GSE53137 and GSE93512, from Gene Expression Omnibus (GEO). Similarly, the top 15 differentially expressed lncRNAs are shown with the heat map (Fig. 1b, c). The shared lncRNAs were analyzed among the three top 15 lists using Venny, and UCA1 was observed to be the most significant among the upregulated lncRNAs (Fig. 1d). Moreover, UCA1 expression level was also evaluated in the cases of GC by interrogating a database containing 407 GC patients from the Cancer Atlas Project (TCGA). As expected, the TCGA GC cohort demonstrated that UCA1 presented the same trend in GC tissues and non-tumor tissues (*P* < 0.001) (Fig. 1e). These results imply that UCA1 overexpression may be useful in the development of a novel prognostic marker for gastric cancer.

### Elevated UCA1 level promoted cell migration and invasion of GC in vitro and in vivo

**Fig. 2:**
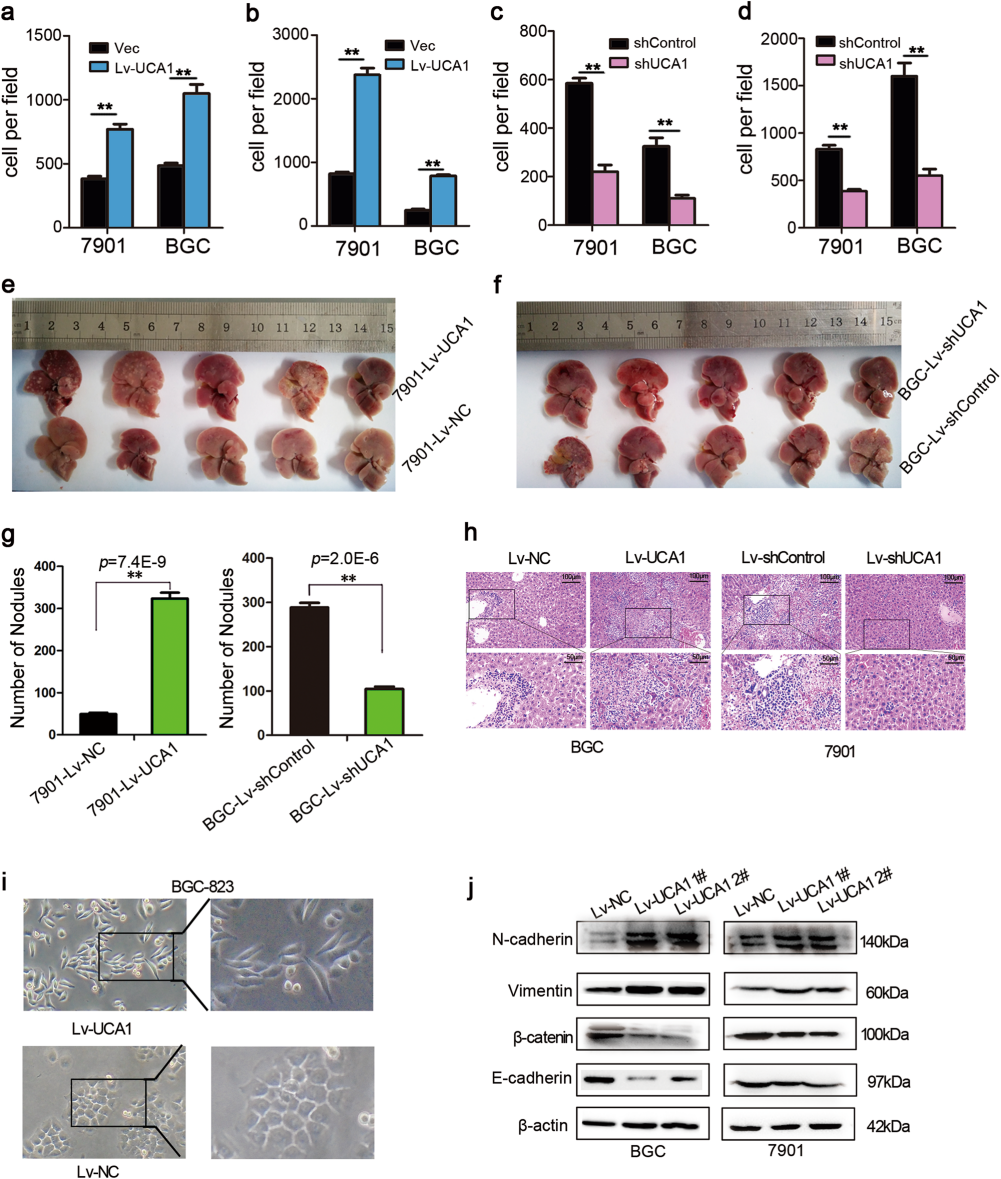
UCA1 promotes cell migration and invasion of GC in vitro and in vivo. **a** The effect of ectopic UCA1 expression in cell migration was assessed by transwell assay in BGC-823 and SGC-7901. The bar plot shows the mean number of migrating cells (±SD) per microscopic field from triplicate samples. **b** The effect of ectopic UCA1 expression in cell invasion was assessed by transwell assay in BGC-823 and SGC-7901. The bar plot shows the mean number of invading cells (±SD) per microscopic field from triplicate samples. **c** The effect of silencing UCA1 expression on cell migration was monitored by transwell assay in BGC-823 and SGC-7901. The bar plot shows the mean number of migrating cells (±SD) per microscopic field from triplicate samples. **d** The effect of silencing UCA1 expression on cell invasion was monitored by transwell assay in BGC-823 and SGC-7901. The bar plot shows the mean number of invading cells (±SD) per microscopic field from triplicate samples. **e**, **f** SGC-7901-Lv-UCA1 and BGC-Lv-shUCA1 stable cells were intravenously injected into BALB/c nude mice via tail vein. After 42 days, the livers were resected. **g** Detectable nodules were quantitatively evaluated on the surface of the livers. Data are expressed as the mean ± SD. (* *p* < 0.05; ** *p* < 0.01; one-way ANOVA). **h** Representative histological photomicrographs of liver tissue sections stained with H&E (scale bars = 50 μm and 100 μm). **i** The cell morphology of BGC-823 cells transfected with Lv-NC and Lv-UCA1 was observed by phase-contrast microscopy. **j** expression level of EMT-related markers was changed in Lv-UCA1 compared with control

To elucidate the functions of UCA1 in GC metastasis in vitro, we examined the effects of UCA1 on GC cell invasion and metastasis by transwell assays and wound healing. The results showed that the migration and invasiveness of GC cells were promoted after overexpression of UCA1 (Fig. 2a, b and Supplementary Fig. S1A) but reduced in the cells with downregulation of UCA1 (Fig. 2c, d and Supplementary Fig. S1B). In the wound-healing experiments, the scratches healed faster in the cells overexpressing UCA1 than in the NC groups (Supplementary Fig. S1C), while the phenomenon was the inverse in the cells with UCA1 shRNA (Supplementary Fig. S1D). These data indicated that UCA1 has the capacity to control a migratory and invasive phenotype in GC cells.

To investigate whether UCA1 affects tumor metastasis in vivo, the different clones of BGC-823 and SGC-7901 cells were inoculated into nude mice by tail-vein injection. At 42 days post injection, the mice were sacrificed to dissect pulmonary and hepatic tissues. The results showed that the 7901-Lv-UCA1 group formed more nodes compared with the 7901-Lv-NC group, while the BGC-Lv-shUCA1 group exhibited less nodes compared to the BGC-Lv-sh control group (Fig. 2e–g), and the nodes were proved to be metastatic tumors by HE staining (Fig. 2h). We also observed that the 7901-Lv-NC group displayed less weight loss compared to the 7901-Lv-UCA1 group (Supplementary Fig. S2A) and the BGC-Lv-shUCA1 group displayed less weight loss compared to the BGC-Lv-sh control group (Supplementary Fig. S2B). Although no obvious nodes were found in the lung tissues from the UCA1 overexpression groups (Supplementary Fig. S3), metastatic tumor cells were observed in the HE-stained pulmonary tissue slides (Supplementary Fig. S4). Taken together, these results indicated that UCA1 possessed the activity to promote hepatic and pulmonary metastases in GC mice.

As shown in Fig. 2i, overexpressing lncRNA-UCA1 in BGC-823 cells showed a mesenchymal-like morphological change, whereas control cells kept the epithelial cell phenotype. The morphological alteration was confirmed by immunostaining using the antibodies against the markers of epithelium (E-cadherin, β-catenin) and mesenchyme (Vimentin, N-cadherin) (Fig. 2j and Supplementary Fig. S5). The data indicated that UCA1 potentially contributes to the tumor metastasis of hepatic and pulmonary tissues during EMT of GC cells.

### UCA1 regulated the expression of ZEB2, which is the EMT-related transcription factor

**Fig. 3:**
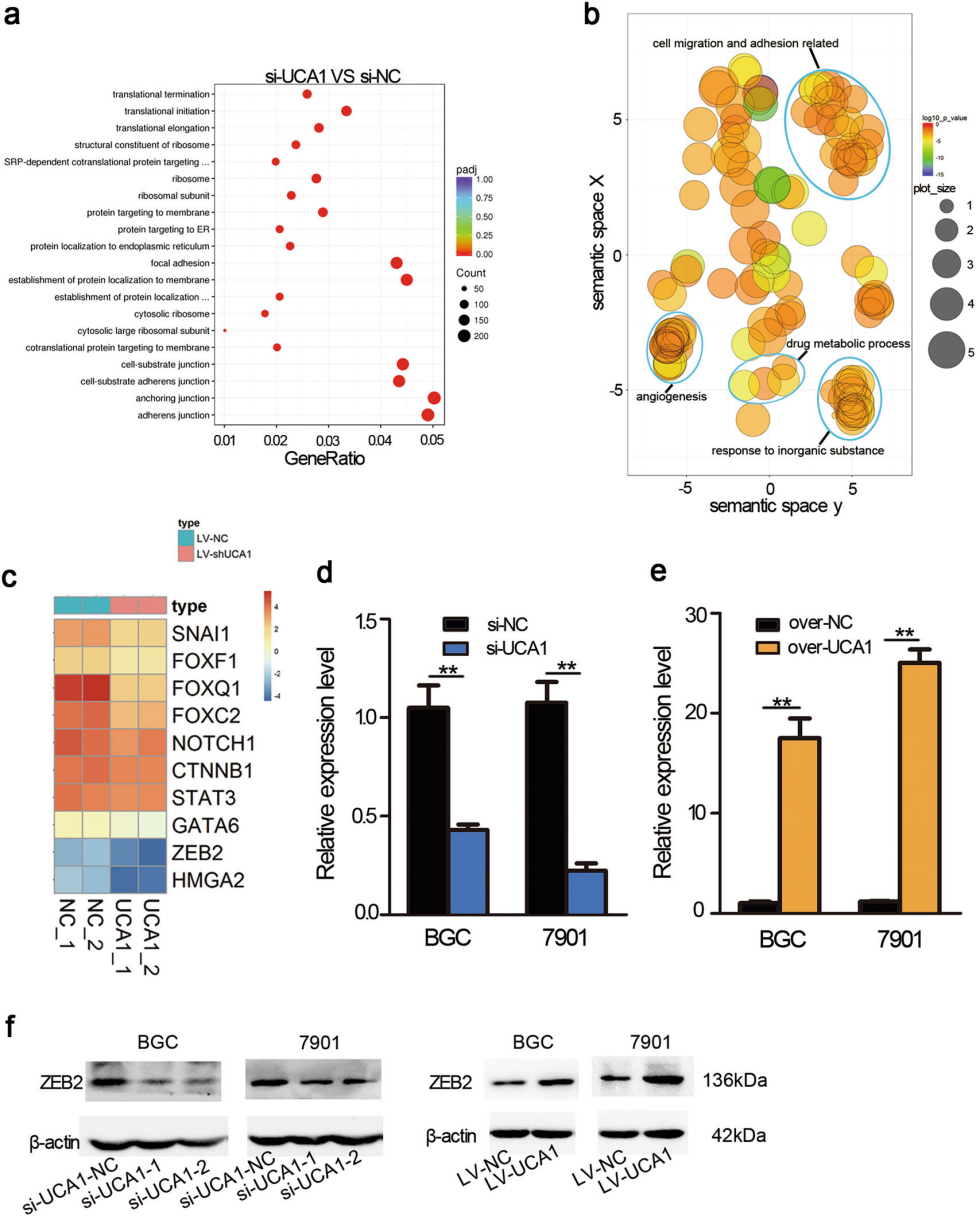
Gene ontology (GO) enrichment analyses of the differentially expressed mRNAs after knocking down UCA1. **a** Gene ontology (GO) analysis of differentially expressed coding genes related to UCA1 knockdown in the biological process categories. The top 20 GO terms are shown. **b** Significantly enriched-GO terms in the biological process category were shown in a two-dimensional semantic space. Color intensity reflects the significance of enrichment test, with dark colors corresponding to lower *P* values and all *P* values lower than 0.05. Circle radiuses depict the sizes of the aggregated-GO terms. **c** Heat map representation of transcription factors involved in EMT-expression levels analyzed by RNA-seq. **d** The relative expression alteration of ZEB2 when UCA1 was knocked down in SGC-7901 and BGC-823. Assays were performed in triplicate, **p < 0.01 (paired Student’s *t*-test). **e** The relative expression alteration of ZEB2 when UCA1 was transfected in SGC-7901 and BGC-823. Assays were performed in triplicate, **p < 0.01 (paired Student’s *t*-test). **f** Western blot was performed to confirm the expression change of ZEB2 when UCA1 is upregulated and downregulated

RNA-seq analysis was performed to examine the gene-expression profiles, when UCA1 was knocked down by the specific shRNA in GC cells. A total of 2562 downregulated genes and 2529 upregulated genes were detected. Gene ontology (GO) analysis revealed that the gene sets were related to focal adhesion, adhesion junction, and anchoring junction (Fig. 3a, b), which suggested that UCA1 could be an important modulator of GC metastasis. Next, we analyzed differentially expressed genes with regard to EMT, which were shown by a heat map. Eleven transcription factor (TF) including ZEB2 were downregulated, when UCA1 was knocked down in SGC-7901 cells (Fig. 3c), which implies that ZEB2 may be a target of UCA1. After overexpression and knockdown of UCA1, qPCR was performed to confirm the RNA-seq data (Fig. 3d, e). As shown in Fig. 3f, depletion of UCA1 significantly reduced the protein expression of ZEB2, whereas UCA1 overexpression enhanced ZEB2 expression. The regulatory relationship between UCA1 and ZEB2 implied that the UCA1–ZEB2 axis may be an important regulatory pathway in the progression of gastric cancer.

### UCA1 sponges directly miR-203 and contributes to GC progression

**Fig. 4:**
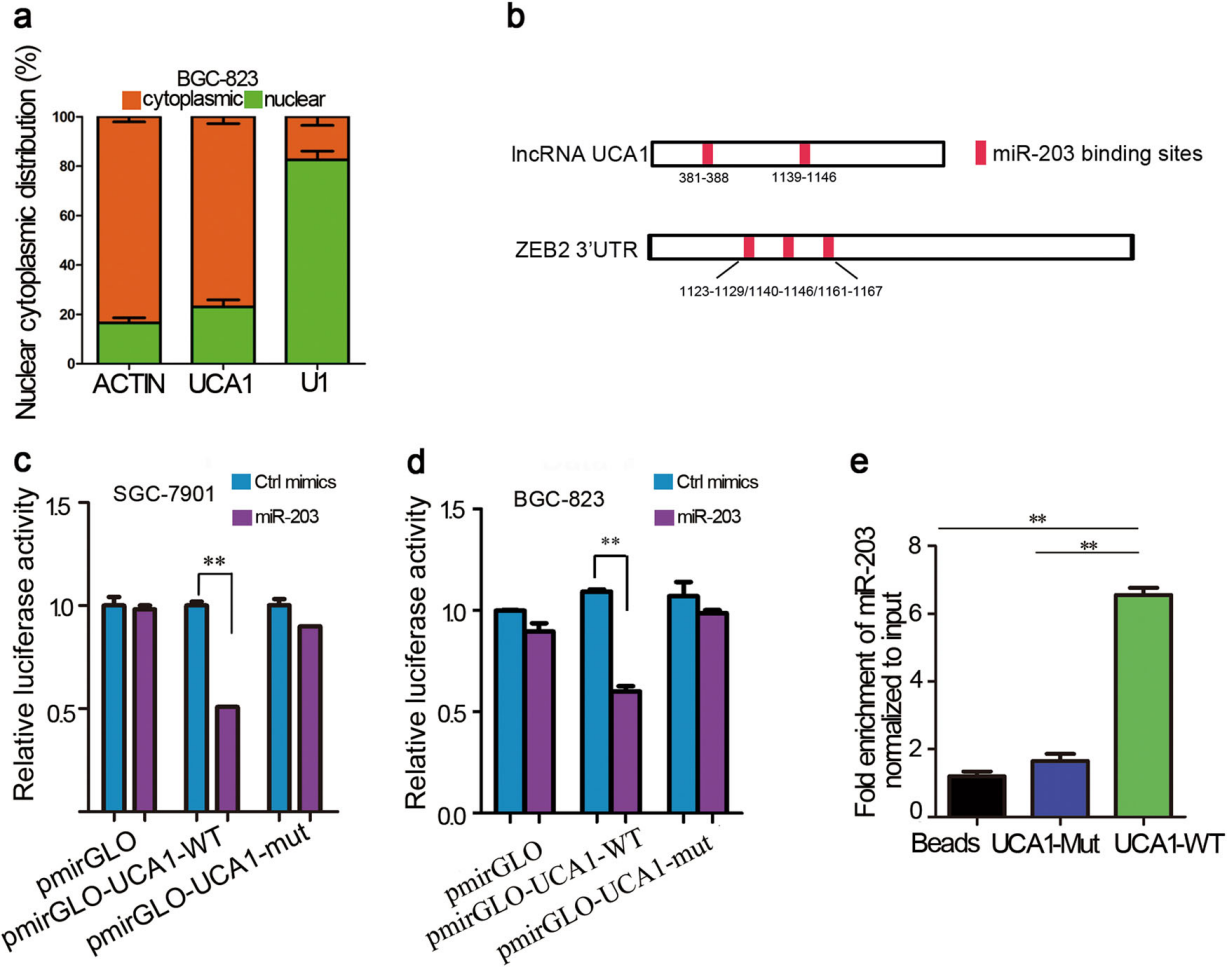
UCA1 is a sponge RNA for miR-203. **a** Cellular fractionation performed in BGC-823 cells followed by RNA isolation and qRT-PCR demonstrates that UCA1 is a cytoplasm-retained lncRNA. β-actin is the cytoplasmic control, and U1 serves as the nuclear control. Error bars indicate S.E.M. Assays were performed in triplicate. **b** Schematic outline of the predicted binding sites of miR-203 on UCA1 and ZEB2. **c**, **d** Relative luciferase activity was performed by dual-luciferase reporter assay. Wild-type (WT) UCA1 cDNA containing putative miR-203 recognition sites or the Mut sequence was cloned in the downstream region of the luciferase gene in the pmirGLO vector. The luciferase-reporter plasmid containing WT or mutant UCA1 was then co-transfected into SGC-7901 and BGC-823 cells along with miR-203 mimics in parallel with Ctrl mimics. Data are expressed as the mean ± SD. Assays were performed in triplicate, **p < 0.01. **e** SGC-7901 cell lysates were incubated with biotin-labeled lncRNA-UCA1; after pull-down, RNA was extracted and miR-203 was assessed by qRT-PCR. Data are expressed as the mean ± SD. Assays were performed in triplicate, **p < 0.01

To explore the molecular mechanisms by which UCA1 performs its biological effects, we isolated the nuclear and cytosolic fractions of UCA1 in BGC-823 cells. As shown in Fig. 4a, the UCA1 transcript was mainly located in the cytoplasm, which suggested that UCA1 might function as a competing endogenous RNA (ceRNA) to sequester miRNAs and eventually lead to the release of corresponding miRNA-targeted transcripts. Thus, we hypothesized that UCA1 contributes to GC progression by functioning as a miRNA sponge. We found that UCA1 contains two direct-binding sites for miR-203 (Fig. 4b).

To explore whether UCA1 sponges miR-203 directly, cDNA sequences with normal UCA1 and mutated miR-203 binding sites were cloned into the downstream region of the luciferase plasmid (named as pmirGLO-UCA1-WT and pmirGLO-UCA1-mut, respectively). The data showed that overexpression of miR-203 reduced the luciferase activities of the WT reporter vectors but not empty vectors or mutant reporter vectors (Fig. 4c, d). To validate the direct binding between miR-203 and lncRNA-UCA1, biotin-labeled pull-down assays were performed. A significant quantity of miR-203 was detected in the UCA1 pull-down pellets compared to the control pellets by qRT-PCR (Fig. 4e). Taken together, these data demonstrated that UCA1 could directly sponge miR-203.

### miR-203 target and suppress ZEB2 expression in an AGO2-dependent manner

**Fig. 5:**
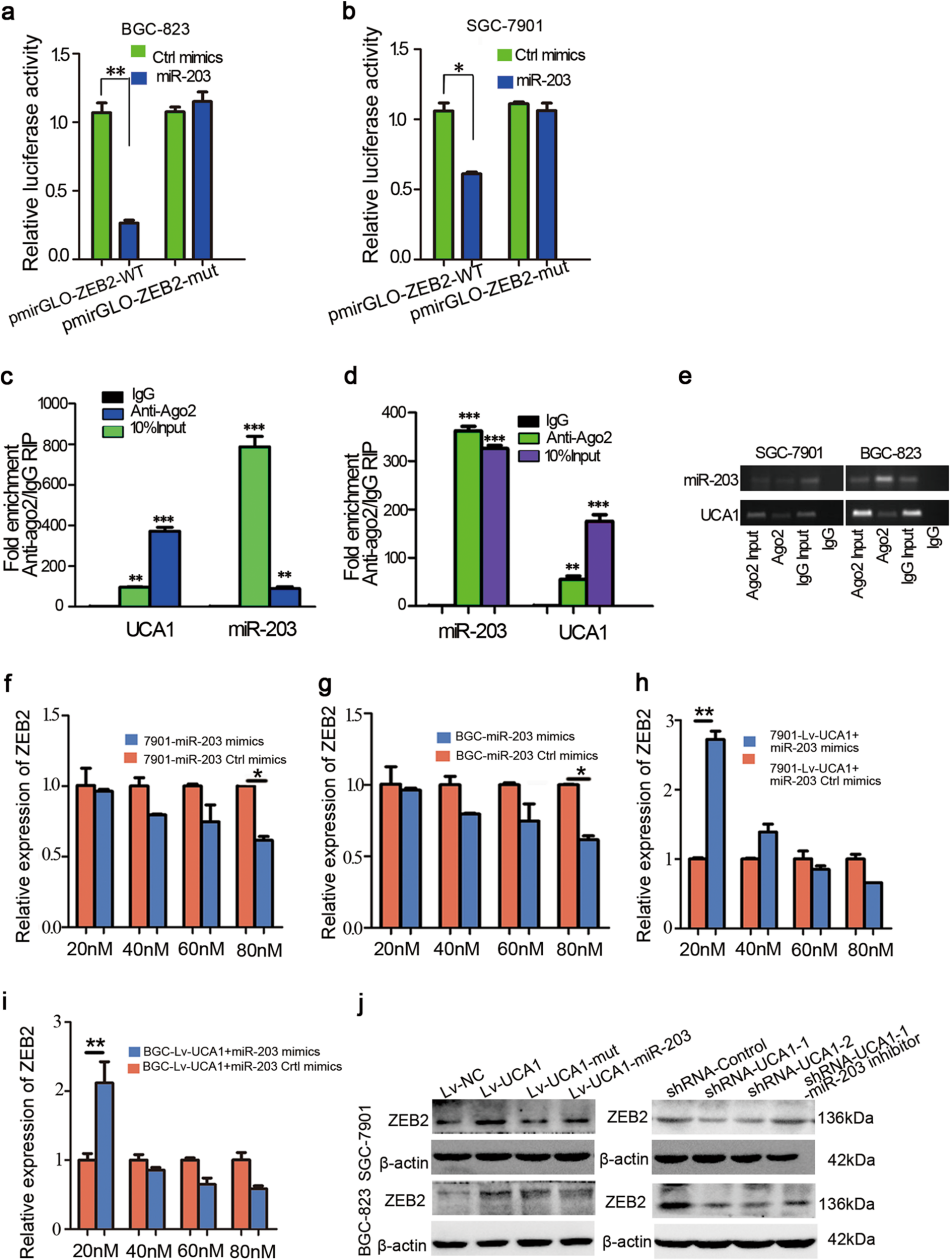
ZEB2 was inhibited by miR-203 in GC cells. **a**, **b** Luciferase reporter plasmids containing WT or mutant ZEB 3′UTR were co-transfected into BGC-823 and SGC-7901 cells with miR-203 mimics in parallel with Ctrl mimics. Each experiment was repeated three times. Data are expressed as the mean ± SD. *p < 0.05 and **p < 0.01. **c**, **d** RIP with mouse monoclonal anti-Ago2, IgG, or 10% input from SGC-7901 and BGC-823 cell extracts. UCA1 and miR-203 expression levels in immunoprecipitates were determined by qRT-PCR. Numbers are mean ± SD. (*n* = 3). **p *<* 0.01 and ***p < 0.001. **e** PCR products of UCA1 and miR-203 were detected by gel electrophoresis. **f**, **g** q-PCR analysis of ZEB2 expression level in SGC-7901 and BGC-823 cells transfected with different doses of miR-203 mimics and control. The error bars in graphs represented SD, and each experiment was repeated three times. *p < 0.05. **h**, **i** q-PCR analysis of ZEB2 expression level in SGC-7901 and BGC-823 stable cells transfected with different doses of miR-203 mimics and control. The error bars in graphs represented SD, and each experiment was repeated three times. *p < 0.05. **j** Western-blot analysis of ZEB2 in BGC-823 and SGC-7901 cells transfected with Lv-control or Lv-UCA1, Lv-UCA1-Mut, and Lv-UCA1 along with miR-203 mimics (Left panel). Western blot to analyze ZEB2 expression level in BGC-823 and SGC-7901 cells transfected with UCA1 siRNA or control along with miR-203 inhibitor (Right panel)

For further study, we found that ZEB2 contains two direct-binding sites for miR-203 in its 3′UTR (Fig. 4b). To prove whether ZEB2 is a target of miR-203 depends on regulation of the ZEB2 3′UTR, we constructed luciferase reporters containing ZEB2 3′UTR-WT or ZEB2 3′UTR-mut, which are pmirGLO-ZEB2-WT or pmirGLO-ZEB2-mut, respectively. The luciferase plasmid (pmirGLO-ZEB2-WT or pmirGLO-ZEB2-mut) was transfected into BGC-823 and SGC-7901 cells. Overexpression of miR-203 decreased the luciferase activity of pmiRGLO-ZEB2-WT; however, mutation of the nucleotides in miR-203′s putative targeting sites resulted in compete abrogation of the repressive effect (Fig. 5a, b). These results suggested that miR-203 exerts its effect on modulating ZEB2 expression by competitively binding ZEB2 3′UTR.

It is well known that microRNAs cause translational repression and/or RNA degradation in an Ago2-dependent manner. To determine whether ZEB2 was regulated by miR-203 in this way, we conducted anti-Ago2 RIP in BGC-823 (Fig. 5c, e) and SGC-7901 cells (Fig. 5d, e). UCA1 was preferentially enriched in Ago2-containing miRNPs compared with control immunoglobulin G (IgG) immunoprecipitates. Similarly, miR-203 was detected at a higher level in Ago2-containing miRNPs than in IgG-containing miRNPs. Thus, UCA1 is abundant in Ago2-containing miRNPs, likely through an association with miR-203. These results indicated that UCA1 and miR-203 both binds with Ago2 in gastric cancer cells.

### UCA1 regulates ZEB2 expression by competitively binding miR-203

Our above findings indicated that UCA1 functions as a ceRNA to regulate ZEB2 expression by sponging miR-203. We then tested whether UCA1 promotes gastric-cancer metastasis in a miR-203/ZEB2-dependent manner. We studied the effect of miR-203 on ZEB2 expression and found that miR-203 inhibited ZEB2 expression in a dose-dependent manner (Fig. 5f, g). Furthermore, we transfected different concentrations of miR-203 into 7901-Lv-UCA1 and BGC-Lv-UCA1 stable cells and observed that ZEB2 expression was first upregulated and then downregulated (Fig. 5h, i). Overexpression of UCA1 WT increased ZEB2 protein expression level, but ectopic expression of miR-203 abrogated this increase (Fig. 5j Left). By using Western blot to analyze the rescue assay by two shRNA of UCA1, we found that deletion of UCA1 decreased ZEB2 expression while inhibiting miR-203 in the shRNA-UCA1 cell line upregulated ZEB2 expression level in BGC-823 and SGC-7901 cells (Fig. 5j Right). These data suggested that UCA1-induced ZEB2 expression depended on the competitive binding with miR-203.

### UCA1 is significantly upregulated in GC and is associated with GC progression

**Fig. 6:**
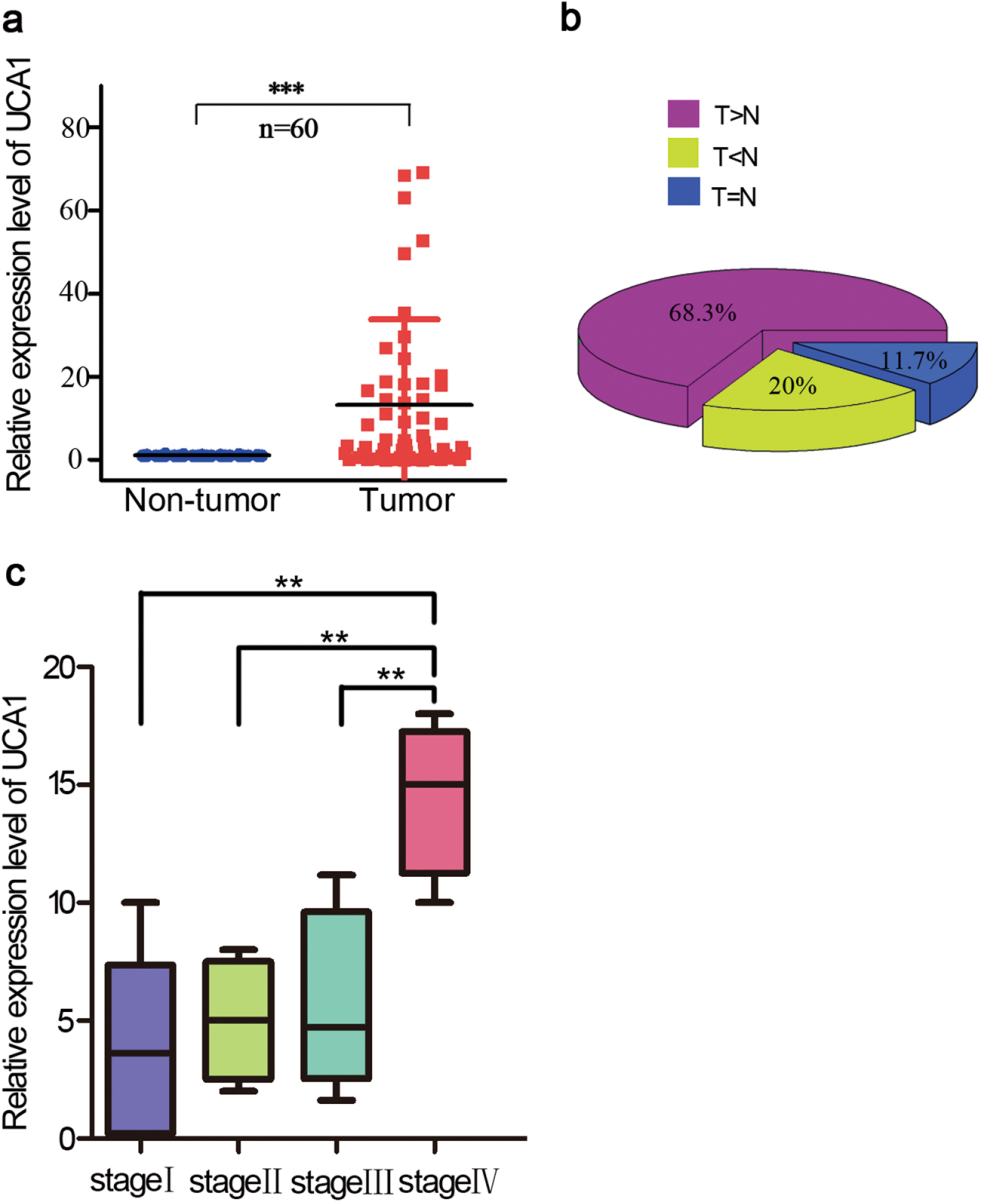
Increased lncRNA UCA1 expression level was detected in GC tissues. **a** Relative expression level of UCA1 was measured by quantitative reverse transcription-PCR in 60 paired gastric tumor tissues and noncancerous normal tissue. ***p *<* 0.001 (paired Student’s *t*-test). **b** The percentage of upregulated UCA1 in GC cases is shown in the pie chart. **c** UCA1 expression was significantly higher in patients with advanced stage

To further define the role of UCA1 in human GC, we measure UCA1 expression level in 60-paired-gastric-cancer samples and adjacent normal tissues by qRT-PCR (Fig. 6a). The results showed significantly higher-UCA1 expression in GC tissues compared to non-tumor tissues. As shown in Fig. 6b, compared to the paired non-tumor tissues, 68.3% (41/60) of GC cases showed increased expression of UCA1 (defined as greater than a two-fold increase). The relationship between UCA1-expression level and clinical-pathological features of 60 paired gastric cancer samples and adjacent normal tissues showed that UCA1 upregulation was closely associated with lymph node metastasis and patient age but not tumor position or patient gender (Supplementary Table 1). We also found that UCA1-expression levels correlated with tumor stage IV in gastric cancer samples (Fig. 6c). This could also explain why high-UCA1-expression levels promote hepatic metastases in GC mice. These results confirmed that UCA1 overexpression is involved in metastasis in GC.

## Discussion

An accumulating amount of evidence indicates that lncRNAs play a critical role in regulating gene expression at epigenetic, transcriptional, and posttranscriptional levels in normal cells^27^. An increasing number of studies suggest that a variety of lncRNAs are frequently aberrantly expressed in cancer cells, exhibiting spatially and temporally regulated expression patterns. The dysregulation of lncRNAs may participate in many key processes in tumor progression, such as cell proliferation^28–31^, differentiation^32–34^, invasion^29,35,36^, metastasis, and death^37–40^. Thus, more efforts should be made to deeply clarify the biological and molecular mechanisms of lncRNAs in cancer.

In this study, we aimed to find the most differentially expressed lncRNA in GC. UCA1 is the most upregulated lncRNA analyzed by our group microarray data and two published GEO datasets in GC. In addition, the higher expression of UCA1 could be detected in the serum of patient with cancer, which suggested that it may be a superior biomarker among current protein-coding biomarkers for cancer diagnostics and prognosis evaluation. In this study, UCA1 expression levels were higher in GC tissues than in the adjacent normal counterparts. GC patients with lymph node metastasis showed significantly higher UCA1 expression levels. Furthermore, we identified the function of UCA1 in GC cells by applying loss- and gain-of-function approaches. Increased UCA1 level promotes cell migration and invasion, and inhibition of UCA1 may be a candidate to suppress cell migration and invasion in tumor progression. The potential of UCA1 in promoting cancer progression has been reported in other cancers. Cai et al.^41^ reported UCA1 promotes gallbladder cancer progression by epigenetically inhibiting E-cadherin expression. Li et al.^42^ reported that dysregulated lncRNA-UCA1 contributes to the progression of gastric cancer through regulation of the PI3K-Akt-mTOR-signaling pathway. Wang et al.^11^ reported that UCA1 promotes tumor metastasis by inducing GRK2 degradation in gastric cancer. Wang et al.^43^ reported that UCA1 promotes cell migration via the miR-216b–FGFR axis in hepatocellular carcinoma. However, epigenetic mechanisms of UCA1 that regulate the metastasis process during gastric cancer have rarely been reported.

Although UCA1 has been shown to play important biological roles in gastric cancer, the precise molecular mechanisms by which UCA1 modulates tumor progression needs to be clarified. UCA1 induces EMT in gastric cancer cells, and many molecules involved in EMT, such as transcription factors and miRNA, have been investigated. The interaction between noncoding RNA, lncRNA, and miRNA in gastric tumor metastasis through modulating EMT remains concealed. With the development of the ceRNA hypothesis, lncRNAs were proposed to communicate with other RNA transcripts through miRNA response elements. In the current study, UCA1, as the most upregulated lncRNA enriched in the cytoplasm, proved for the first time to act as a sponge lncRNA to promote metastasis in GC cells.

In the present research, luciferase and RNA pull-down assays showed that UCA1 is a direct target of miR-203, and it acts as a sponge for miR-203. Previous reports proved that miR-203 possesses a tumor suppressive role in many cancers, including lung cancer, GC, breast cancer, HCC, and colorectal cancer, and miR-203 regulates a cohort of metastatic genes including ZEB2 in prostatic carcinoma^44–46^. ZEB2 is known as a crucial inducer during EMT in gastric adenocarcinoma^47^, which contributes to the loss of epithelial marker E-cadherin and disrupts cell-to-cell adhesion. In addition, ZEB2 elevated mesenchymal markers, as well as facilitated tumor cell invasion^48,49^. In our findings, we proved that miR-203 inhibit ZEB2 by directly targeting the 3′-UTR of ZEB2 by luciferase and q-PCR assays. Moreover, we verified that miR-203 inhibit ZEB2 in an Ago-2-dependent manner by a RIP assay.

**Fig. 7:**
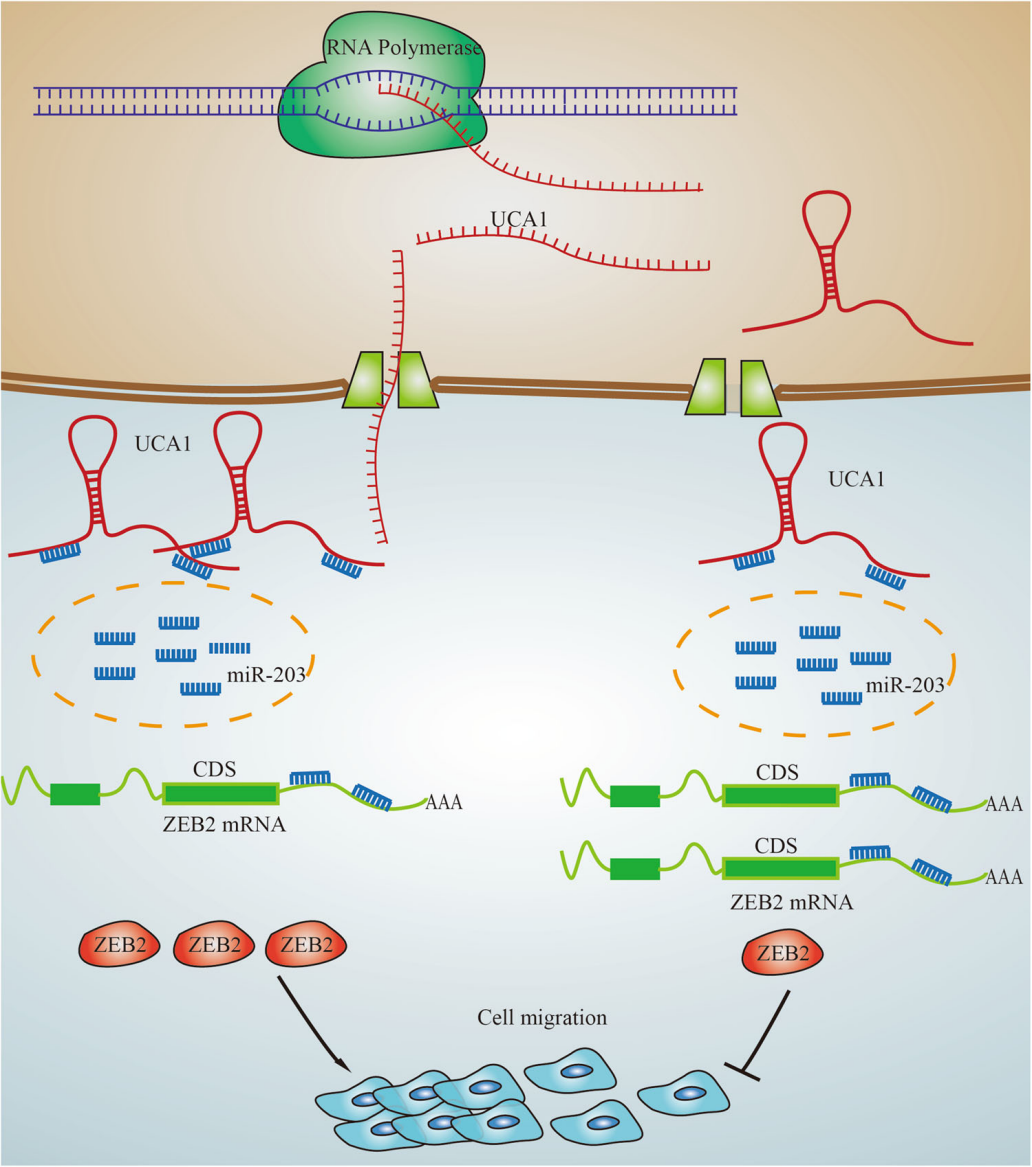
Schematic for the regulatory relationship among UCA1, miR-203, and ZEB2 in GC cells

We revealed that UCA1 competitively binds to miR-203 and subsequently increases the expression level of its target gene ZEB2 to promote the migration and invasion of GC cells. Furthermore, UCA1 functions as a ceRNA, regulating ZEB2 expression by competitively binding miR-203 and then inducing EMT in GC (Fig. 7). Although the mechanism of lncRNA as ceRNA to regulate coding gene has been mentioned in several tumorigenesis, our finding that UCA1 sponged miR-203 to regulate ZEB2 is still a novel contribution to understanding the metastasis of GC. Collectively, the present study implies that the UCA1/miR-203/ZEB2 regulatory network might be a potential therapeutic target for the highly aggressive GC.

## Materials and methods

### Tissue collection

Fresh-frozen and paraffin-embedded gastric cancer tissues and corresponding adjacent non-tumor samples were obtained from Chinese patients at the 3rd Affiliated Hospital of Harbin between 2006 and 2008. All cases were reviewed by a pathologist and histologically confirmed as GC (stages II, III, IV; 7th Edition AJCC) based on histopathological evaluation. Clinical pathology information was available for all samples. No local or systemic treatment was conducted in these patients before the operation. The study was approved by the Research Ethics Committee of Southeast University, China. Informed consents were obtained from all the patients.

### Cell culture

GC cell lines (BGC-823, SGC-790) were purchased from the Institute of Biochemistry and Cell Biology at the Chinese Academy of Science (Shanghai, China). Cells were cultured in RPMI 1640 medium supplemented with 10% fetal bovine serum at 37 °C in an atmosphere containing 5% CO_2_.

### RNA extraction and qRT-PCR analyses

Total RNA was extracted from tissues or cultured cells using TRIzol reagent (Invitrogen, Carlsbad, CA). For qRT-PCR, RNA was reverse transcribed to cDNA by using a reverse transcription kit (Takara, Dalian, China). The ABI7900 real-time system (Applied Biosystems, USA) was utilized for quantitative reverse transcriptase-PCR reaction. The gene-specific primers are shown in Supplementary Table 2. β-actin was employed as a loading control for mRNA and lncRNA. For microRNA analysis, real-time qPCR was performed as above, and the relative expression of RNAs was calculated using the comparative Ct method.

### RNA binding protein immunoprecipitation (RIP) assay

RNA immunoprecipitation was performed following the manufacturer’s protocol with the EZ-Magna RIP kit (Millipore, Billerica, MA, USA). The cells at 80–90% confluency were scraped off and lysed in complete RIP-lysis buffer. Then, 100 μL of whole-cell extract was incubated with RIP buffer containing magnetic beads conjugated with human anti-Ago2 antibody (Millipore), while the negative control was incubated with normal mouse IgG (Millipore). Anti-SNRNP70 (Millipore) was used as a positive control for the RIP procedure. The samples were incubated with Proteinase K to digest the proteins, and subsequently, immunoprecipitated RNA was isolated. The RNA concentration was measured using a NanoDrop (Thermo Scientific, USA), and the RNA quality was assessed by a bioanalyzer (Agilent, Santa Clara, CA, USA). At last, purified RNA was subjected to qRT-PCR analysis to demonstrate the presence of the binding targets using the specific primers.

### Western blot analysis

Total cell lysates were prepared in a 1 × sodium dodecyl sulfate buffer. Identical quantities of proteins were separated by sodium dodecyl sulfate-polyacrylamide gel electrophoresis and transferred onto nitrocellulose-filter membranes. After an incubation with the antibody against ZEB2 (Sigma-Aldrich, USA), E-cadherin(cell Signaling Technology, USA), N-cadherin(cell Signaling Technology, USA), Vimentin(cell Signaling Technology, USA), β-catenin(Santa Cruz, USA), and β-actin (Sigma-Aldrich, USA), the blots were treated with IRdye 800-conjugated goat anti-rabbit IgG or IRdye 700-conjugated goat anti-mouse IgG. The blots were evaluated by an Odyssey infrared scanner (Li-Cor). β-actin was used as a loading control.

### Biotin-labeled miR-203 capture

Cells were harvested 24 h after transfection and then lysed on ice for 30 min in 250 μL of cell-lysis buffer (10 mM KCl, 1.5 mM MgCl2, 10 mM Tris-HCl pH 7.5, and 5 mM dithiothreitol) containing RNasin (Takara, Japan) and proteinase inhibitor cocktail (Roche, Sweden). The supernatants were centrifuged for 5 min at 12,000 × g and then 500 μL of NaCl (1 M) and 30 μL of beads (Dynabeads MyOne Streptavidin C1; Life Technologies, USA) were added. Before being added to the supernatant, the beads were washed five times with solution A (0.1 M NaOH, 0.05 M NaCl), then blocked with 1 mg/mL BSA (Roche) and 1 mg/mL yeast tRNA (Ambion, USA) overnight. Afterwards, the beads were cleaned using washing buffer (5 mM Tris-HCl pH 7.5, 0.5 mM EDTA, 1 M NaCl) and the lysate was kept at 4 ℃ for 4 h. RNA was extracted from the remaining beads with TRIzol Reagent (Life Technologies, USA) and evaluated by real-time PCR assay. The entire PCR assay is the same as the previous real-time PCR protocol.

### Luciferase reporter assay

PmirGLO, pmirGLO-UCA1-WT, or pmirGLO-ATB-mut(miR-203) was co-transfected with miR-203 mimics or miR NC into cells by Lipofectamine-mediated gene transfer. pmirGLO, pmirGLO-ZEB2-WT, or pmirGLO-ZEB2-mut(miR-203) was transfected into the cells by lipofectamine-mediated gene transfer. The relative luciferase activity was normalized to Renilla luciferase activity 48 h after transfection.

### Vector construction, lentiviral construction, and cell transfections

The cDNA encoding lncRNA-UCA1 was amplified by the *Pfu* Ultra II Fusion HS DNA Polymerase (Stratagene, Agilent Technologies, USA) and subcloned into the Nhel and XhoI sites of the pcDNA3.1 vector (Invitrogen, USA) and named pcDNA3.1-UCA1. The full length of lncRNA-UCA1-WT or lncRNA-UCA1-mut (miR-203) was amplified using PCR and subcloned into the pmirGLO vector (Promega, Madison, WI, USA) for the Luciferase-reporter assay using the one-step directed cloning kit (Novoprotein, Shanghai, China). The 3′ untranslated regions (3′-UTR) of ZEB2 mRNA containing the intact miR-203 recognition sequences were PCR amplified and subcloned into the *Sac I* and *Sal II* sites of the pmirGLO vector.

Lentiviral vector expressing UCA1, shUCA1, or an empty lentiviral vector was purchased from GeneChem (Shanghai, China). Cell infection was performed following the manufacturer’s protocol. Cells stably overexpressing UCA1, shUCA1, or the empty vector were selected by puromycin (2 µg/ml).

### Microarray analysis

The total RNA was extracted from a mixture of five paired tumor tissues and noncancerous normal tissue, respectively, and it was amplified and transcribed into fluorescent cRNA using the Quick Amp-Labeling kit (Agilent Technologies, Palo Alto, CA, USA). The labeled cRNA was then hybridized onto the Human LncRNA Array v2.0 (8 × 60 K, ArrayStar, Rockville, MD, USA), and the arrays were scanned by the Agilent Scanner G2505B and analyzed with Agilent Feature Extraction software (Version 10.7.3.1). Quantile normalization and subsequent data processing were performed using the GeneSpring GX v11.5.1 software package (Agilent Technologies, USA). The differentially expressed lncRNAs with statistical significance were identified using volcano plot filtering. Fold change ≥ 2 and a *P*-value ≤ 0.05 were defined as the threshold.

### Cell migration and invasion assays

At 48 h after transfection, the cells in serum-free media were placed into the upper chamber of an insert for migration assays (8-μm pore size, Millipore) and invasion assays with Matrigel (Sigma-Aldrich, USA). Media containing 10% FBS was added to the lower chamber. After incubation, the cells that had migrated or invaded through the membrane were stained with methanol and 0.1% crystal violet, imaged, and counted using an IX71 inverted microscope (Olympus, Tokyo, Japan).

### TCGA data analysis

Processed TCGA expression data were downloaded from the TCGA official website (http://cancergenome.nih.gov/), and the upper quantile normalized FPKM (fragments per kilobases per million) values were used. Then, the expression data were log_2_-transformed. Raw data from sets GSE53137 and GSE93512, generated using the Arraystar Human LncRNA microarray V2.0 platform, were downloaded from the GEO database.

### Metastasis assay in vivo

Female BALB/c mice were randomly divided into four groups. A total of 2 × 10^6^ cells transfected with sh-UCA1 in BGC and over-UCA1 in SGC-7901 wild type or the negative control were injected into each of 4–5-week-old female BALB/C nude mice through the tail veins. The mice were housed for six weeks following the injection and were then euthanized by cervical dislocation. The lungs and livers were dissected and examined histologically. Briefly, the tissues were first fixed with 10% formalin, embedded in paraffin, and then stained with hematoxylin and eosin. The histological changes of the tissues were observed under a light microscope, and the number of metastases was counted.

### Statistical analysis

Student’s *t*-test (two-tailed), one-way ANOVA and the Mann–Whitney test were performed using *SPSS 16.0* software. *P* values less than 0.05 were considered significant. Differentially expressed gene analysis was performed with R (https://www.r-project.org/, v3.0.1) and R packages. Normalization of Arraystar Human LncRNA microarray data was implemented with the R package “limma”.

## Electronic supplementary material

Supplementary figure legends

Supplementary figures

Supplementary table 2

Supplementary table 1
