# Supplementary material for: LncRNA UCA1 promotes tumor metastasis by inducing miR-203/ZEB2 axis in gastric cancer
Source: Cell Death Dis. 2018 Nov 21;9(12):1158. doi: 10.1038/s41419-018-1170-0 (PMC6249325; doi:10.1038/s41419-018-1170-0)
Supplement: Supplementary file 1 — Supplementary figure legends [file 41419_2018_1170_MOESM1_ESM.pdf]

Supplementary Figure Legends

UCA1 sponges miR-203 to promote metastasis of gastric cancer cells through the upregulation of ZEB2

Figure S1 UCA1 promotes cell migration and invasion of GC *in vitro*

A: The effect of ectopic UCA1 expression in cell migration and invasion was assessed by transwell assay in BGC-823 and SGC-7901.

B: The effect of silencing UCA1 expression on cell migration and invasion was monitored by transwell assay in BGC-823 and SGC-7901.

C: Wouding healing assays showed the migration ability of SGC-7901 and BGC-823 was increased in the over-UCA1 group than in the NC group.

D: Wouding healing assays showed the migration ability of SGC-7901 and BGC-823 was decreased in the si-UCA1 group than in the NC.

Figure S2 The altered weight of nude mice

A and B: The altered weight of nude mice is different between two groups, intravenously injected with SGC-7901-Lv-UCA1 and BGC-Lv-shRNAUCA1 for 42 days. Data are expressed as the mean ± SD. $*p < 0.05$.

Figure S3 SGC-7901-Lv-UCA1 and BGC-Lv-shRNA-UCA1 stable cells were intravenously injected into BALB/c nude mice via tail vein. After 42 days, lungs were resected and quantitatively evaluated detectable nodules on the surface of the livers.

Figure S4 Representative histological photomicrographs of lung tissue sections stained with H&E (10×and 40×).

Figure S5 The expression change of CDH1 when UCA1 is up-regulated and down-regulated in GC cells.

A and B: q-PCR was performed to confirm the expression change of CDH1 when UCA1 is down-regulated. Assays were performed in triplicate, $**p < 0.01$ (paired Student's t test).

C and D: q-PCR was performed to confirm the expression change of CDH1 when UCA1 is up-regulated. Assays were performed in triplicate, $**p < 0.0$ (paired Student's t test).

E: Western Blot was performed to confirm the expression change of CDH1 when UCA1 is up-regulated and down-regulated in GC cells.
