# Supplementary material for: LncRNA UCA1 promotes tumor metastasis by inducing miR-203/ZEB2 axis in gastric cancer
Source: Cell Death Dis. 2018 Nov 21;9(12):1158. doi: 10.1038/s41419-018-1170-0 (PMC6249325; doi:10.1038/s41419-018-1170-0)

Supplementary Fig. S1

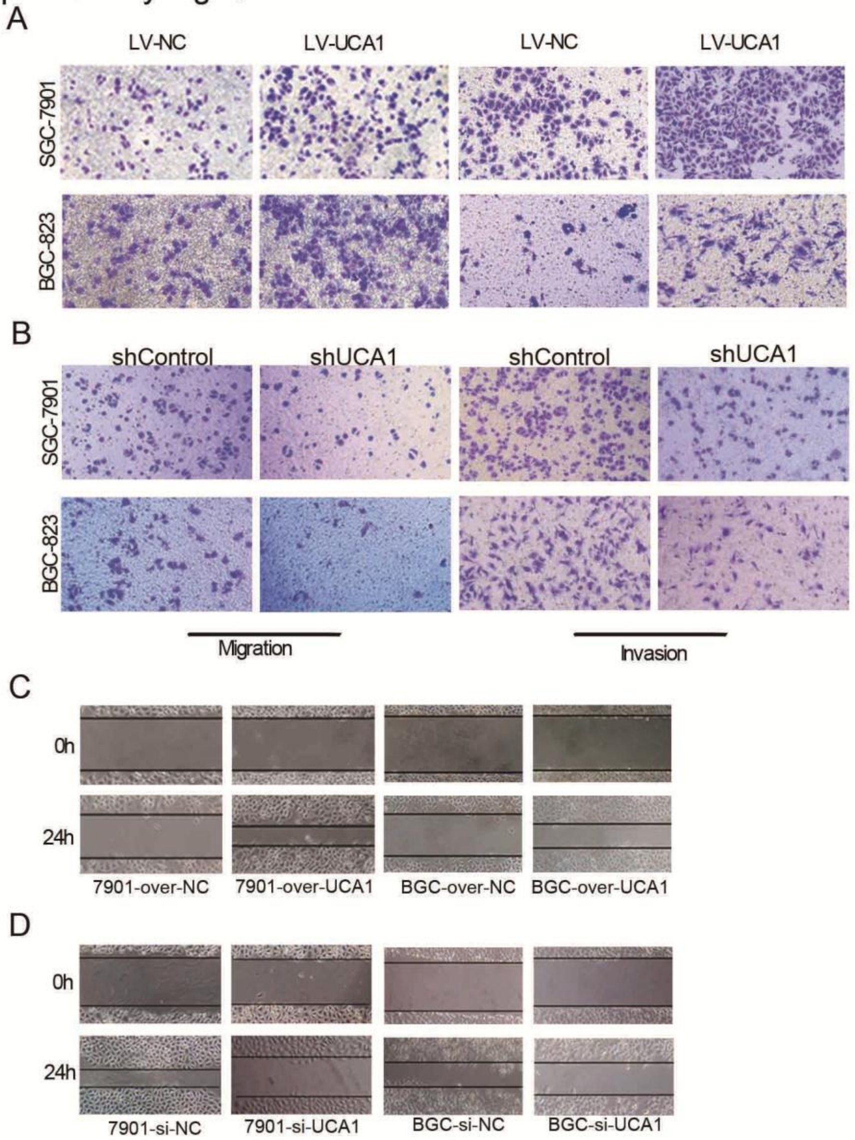

Supplementary Fig. S2

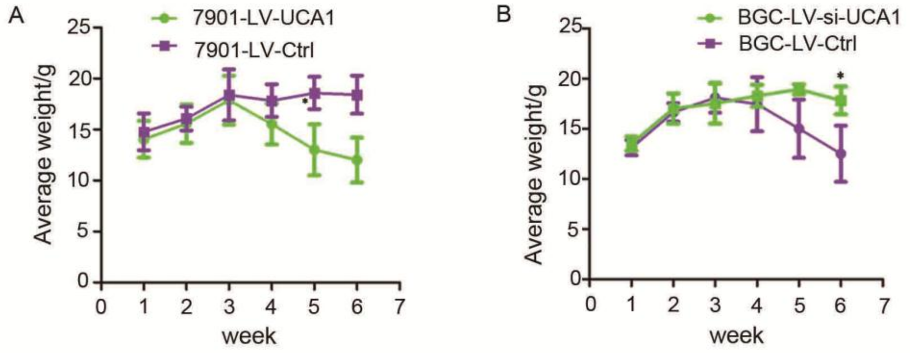

supplementary Fig. S3

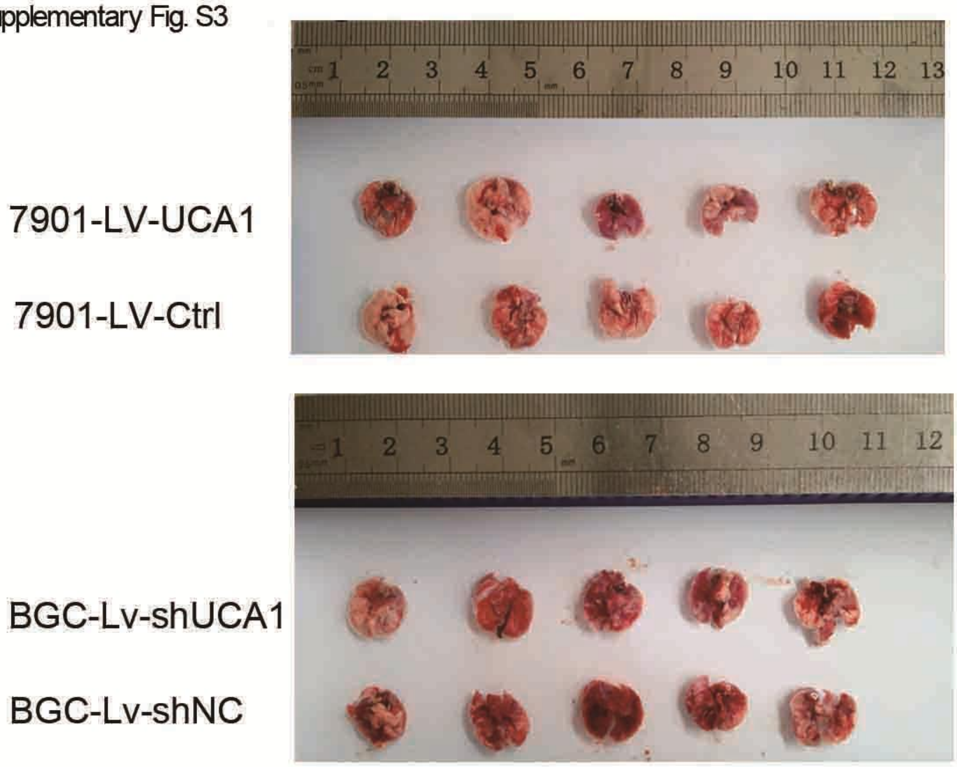

Supplementary Fig. S4

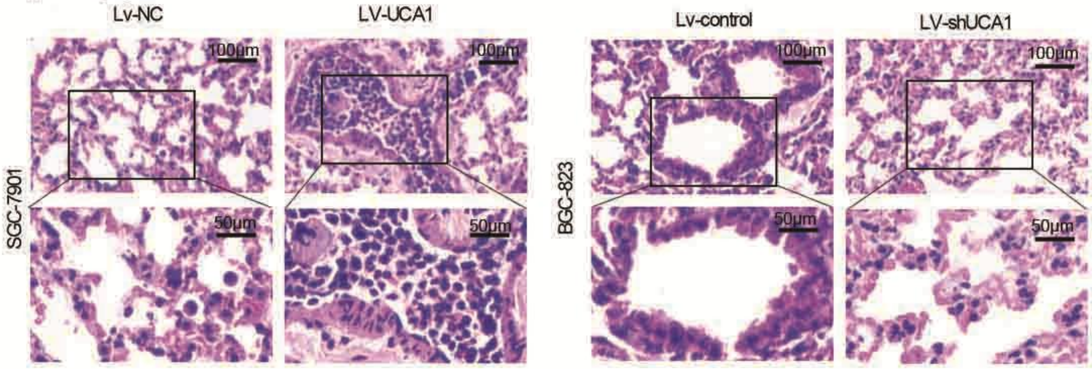

Supplementary Fig. S5

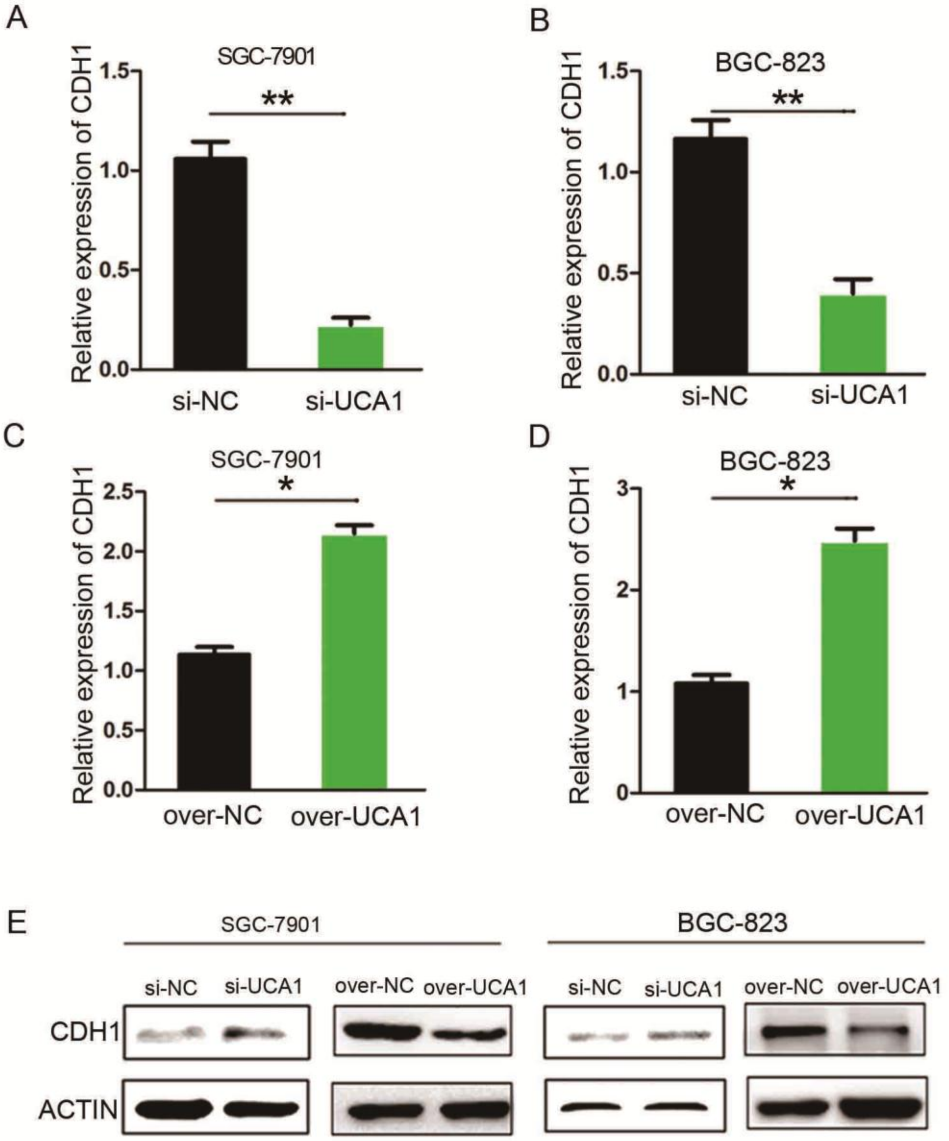

Supplement: Supplementary file 2 — Supplementary figures [file 41419_2018_1170_MOESM2_ESM.pdf]
