# Supplementary material for: LncRNA UCA1 promotes tumor metastasis by inducing miR-203/ZEB2 axis in gastric cancer
Source: Cell Death Dis. 2018 Nov 21;9(12):1158. doi: 10.1038/s41419-018-1170-0 (PMC6249325; doi:10.1038/s41419-018-1170-0)
Supplement: Supplementary file 3 — Supplementary table 2 [file 41419_2018_1170_MOESM3_ESM.pdf]

Supplementary Table 2. Oligonucleotide Sequences used in this study

Gene	Sequence (5′to 3′)	Products size (bp)
ACTB	F:GTCATTCCAAATATGAGATGCGT	125
	R:GCTATCACCTCCCCTGTGTG	
UCA1	F:CTCTCCATTGGGTTCACCATTC	254
	R:GCGGCAGGTCTTAAGAGATGAG	
UCA1 clone	F:CCCAAGCTTTGACATTCTTCTGGAC	1423
	R:GGAATTCAATCAGGCATATTAGCTT	
ZEB2	F: CAGACCGCAATTAACAATGG	155
	R: TACTCCTCGATGCTGACTGC	
siRNA sequence		
UCA1-siRNA1	Sense:GUUAAUCCAGGAGACAAAGATT	
	Antisense:UCUUUGUCUCCUGGAUUAACTT	
UCA1-siRNA2	Sense: GGACAACAGUACACGCAUAdTdT	
	Antisense:TATGCGTGTACTGTTGTCCdTdT	
