# Supplementary material for: LncRNA UCA1 promotes tumor metastasis by inducing miR-203/ZEB2 axis in gastric cancer
Source: Cell Death Dis. 2018 Nov 21;9(12):1158. doi: 10.1038/s41419-018-1170-0 (PMC6249325; doi:10.1038/s41419-018-1170-0)
Supplement: Supplementary file 4 — Supplementary table 1 [file 41419_2018_1170_MOESM4_ESM.pdf]

Supplementary Table 1. Clinicopathological correlation of UCA1 expression in GC cases

Characteristics	N(%)	UCA1		*p*
		High	Low	Chi-squared
**Gender**				
Male	36(75%)	26	10	1.000
F**emale**	12(25%)	8	4	
**Age**				
＜65	37（77%)）	26	11	0.027*
≥65	11（23%)）	3	8	
**Tumor size**				
＜5cm	31（65%)）	23	8	0.719
≥5cm	17（35%）	11	6	
**Differentiation**				
Low	31（65%)）	20	11	0.333
Middle	17（35%）	14	3	
**Lymph node metastasis**				
Negative	9(19%)	3	6	0.032*
Positive	39(81%)	30	9	
**HP infection**				
Negative	23(48%)	18	5	0.442
Positive	25(52%)	16	9	
**Vessel invasion**				
Negative	38(79%)	26	12	0.745
Positive	10(20%)	8	2	

*chi-square test, *＜0.05.
